# Transversus Abdominis Plane Block Reduced Early Postoperative Pain after Robot-assisted Prostatectomy: a Randomized Controlled Trial

**DOI:** 10.1038/s41598-020-60687-y

**Published:** 2020-02-28

**Authors:** Hideki Taninishi, Takashi Matsusaki, Hiroshi Morimatsu

**Affiliations:** Department of Anesthesiology and Resuscitology, Okayama University Graduate School of Medicine, Dentistry and Pharmaceutical Sciences, 2-5-1, Shikata-Cho, Kitaku, Okayama City, 700-8558 Japan

**Keywords:** Randomized controlled trials, Outcomes research

## Abstract

Analgesic effect of transversus abdominis plane block (TAP block) in lower major abdominal laparoscopic surgery with about 5 cm of maximum surgical scar has been controversial. We hypothesized that TAP block has benefits, so the analgesic effect of TAP block after robot-assisted laparoscopic prostatectomy (RALP) was evaluated. One hundred patients were enrolled in this prospective, double-blinded, randomized study. Standardized general anesthesia with wound infiltration on camera port and fentanyl dose limit of 3 µg/kg was provided. Ultrasound-guided, single-shot subcostal TAP block with either 0.375% ropivacaine (Ropivacaine group, 48 patients) or normal saline (Control group, 52 patients) was performed by anesthesiologist in charge (34 anesthesiologists) after surgical procedure. Pain score using numerical rating scale (NRS) and postoperative intravenous fentanyl were evaluated for the first 24 postoperative hours. Median values (interquartile range) of NRS scores when the patients were transferred to post-anesthesia care unit (PACU) were 5 (2–7) in Ropivacaine group and 6 (4–8) in Control group at rest (P = 0.03), 5 (2–8) in Ropivacaine group and 7 (5–8) in Control group during movement (P < 0.01). These significant differences disappeared at the time of discharging PACU. Fentanyl doses for the first 24 postoperative hours were 210 µg (120–360) in Ropivacaine group and 200 µg (120–370) in Control group (P = 0.79). These results indicated that subcostal TAP block by anesthesiologists of varied level of training reduced postoperative pain immediate after RALP. TAP block had fundamental analgesic effect, but this benefit was too small to reduce postoperative 24-hour fentanyl consumption.

## Introduction

In the past few decades, lower abdominal major surgical procedures such as colectomy, hysterectomy and prostatectomy have changed from laparotomy to laparoscopic methods. Although epidural anesthesia has been used as gold standard in laparotomy, smaller surgical scar in laparoscopic procedures and early recovery after surgery motivated us to consider peripheral nerve blocks instead of epidural anesthesia^1,2^.

Transversus abdominis plane block (TAP block), first reported in 2001^3^, has recently been spreading as an alternative to epidural anesthesia. Analgesic effect of TAP block has been established in laparoscopic cholecystectomy^4–6^. Although enhanced recovery after surgery (ERAS) protocol in elective colorectal surgery recommended abdominal wall blocks because of postoperative less opioid use and early recovery^2^,decrease in pain score by TAP block in lower major abdominal laparoscopic procedures remains controversial^7–10^. One of reasons for this controversy is these major procedures require a larger port scar in order to extract resected organs. Furthermore, there are several technical biases affecting TAP block efficacy such as approach options, timing of TAP block (preoperative or postoperative), local anesthetic concentration and dose, and supplementary analgesics. It is necessary to minimize these biases when evaluating the accurate analgesic effect of TAP block.

We hypothesized that TAP block in lower major abdominal laparoscopic surgery reduces postoperative pain. We obtained data for cases of robot-assisted laparoscopic prostatectomy (RALP) because we have enough number of patients, maximum surgical scar is ≈5 cm as colectomy, degrees of surgical invasion for patients are equivalent because of small bias of surgical skills between surgeons by robot assistance. The primary objective of this study was postoperative pain when the patients transferred to post-anesthesia care unit (PACU) both at rest and during movement, and the secondary objective was opioid consumption during the first 24-hour period after RALP.

## Methods

This study was a prospective, randomized, double-blinded, placebo-controlled in Okayama University Hospital. All methods in this study were performed in accordance with the relevant guidelines (Declaration of Helsinki), and study approval was obtained from Okayama University Institutional Review Board (Number 1702-005, 1902-034 for additional perioperative variables of remifentanil and postoperative nausea vomiting (PONV)). This trial was registered prior to first patient enrollment at http://www.umin.ac.jp/english/ (UMIN 000024632, registered on October 30, 2016). This study was reported according to the Consolidated Standards of Reporting Trials (CONSORT) guidelines^11^. Male patients scheduled for RALP by prostate cancer had eligibility. Patients with American Society of Anesthesiology grade of 3 or higher, patients with regular use of opioids, and patients with body weight of less than 40 kg were not enrolled. Patients were enrolled consecutively after obtaining written informed consent at the time of preoperative evaluation.

### Anesthesia protocol

**Figure 1:**
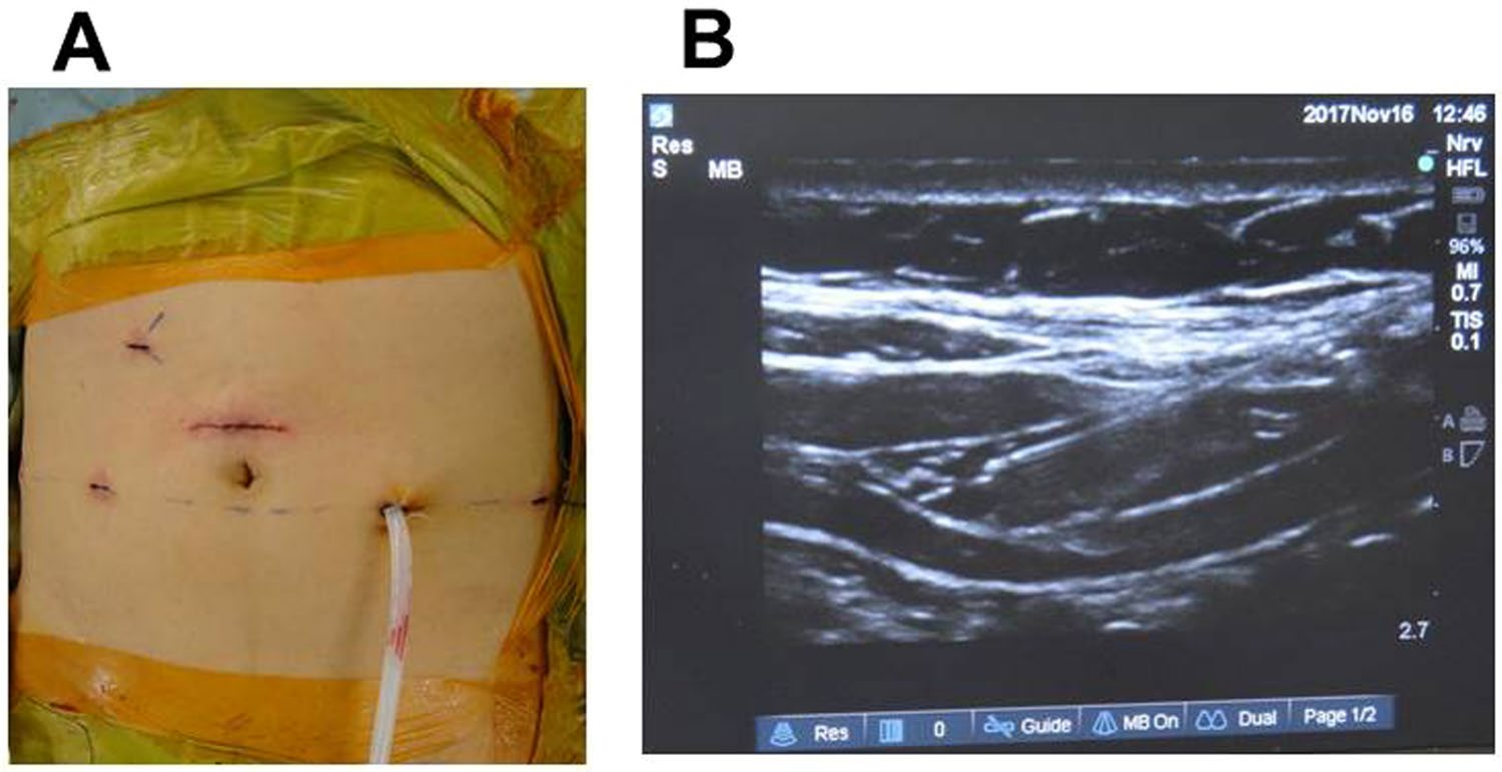
(**A**) Typical operation scar of RALP. The camera port scar was located 2–3 cm above umbilicus. This scar was initially 12 mm in diameter during the laparoscopic procedure and was then enlarged to about 5 cm in order to extract the prostate. (**B**) Ultrasound view during subcostal TAP block. The needle was inserted from the lateral end of the rectus sheath muscle (right side of the screen), and the drug was spread on the transversus abdominis plane from the medial end of the internal abdominal oblique muscle to outward direction.

No premedication drug was given. Anesthesia induction was performed by infusion of propofol (1~1.5 mg/kg) and rocuronium in 100% oxygen. After tracheal intubation, anesthesia was maintained by desflurane, remifentanil and fentanyl in 40% oxygen. Intravenous fentanyl during surgery could be used with a limit of approximately 3 µg/kg of body weight. There was no limit for the dose of remifentanil during surgery, but remifentanil infusion had to terminate before completion of surgery. After completion of laparoscopic procedure, we asked surgeons to give 10 ml of 0.2% ropivacaine only in the camera port scar (≈5 cm in length (Fig. 1A)), and no additional intravenous fentanyl was allowed after this wound infiltration. No other analgesic drug was allowed until completion of the first interview in post-anesthesia care unit (PACU).

### Group assignment and drug preparation for TAP block

The patients were assigned to either one of two groups, a group in which 15 ml of 0.375% ropivacaine was given on each side (total of 30 ml) (Ropivacaine group) or a group in which 15 ml of 0.9% saline was given on each side (total of 30 ml) (Control group). We did not set group without TAP block in order to keep blindness to TAP block practitioners. A computer randomization was carried by a person (an assigner) who was blinded to this study and was stored in a computer that no researcher could access.

After induction of general anesthesia, a third-party anesthesiologist without relation to the patients asked the assigner about group assignment. The anesthesiologist prepared two preparations of 20 ml of 0.375% ropivacaine (Ropivacaine group) or 20 ml of 0.9% saline (Control group) for bilateral TAP block according to the assignment. The drugs were labeled with the study enrollment number (such as TAP-*XX*), so nobody could know the assignment except for the third-party anesthesiologist.

### TAP block and data collection at the PACU

When the surgery was completed, bilateral TAP block was performed by the anesthesiologist in charge of the patient. Since the camera port was located above the umbilicus (Fig. 1A, may be innervated by Th9), a subcostal approach was employed. A 21-gauge needle of 100 mm in length (Stimuplex A, BBraun Inc. Melsungen, Germany) was inserted from bilateral lateral ends of rectus abdominis muscles using ultrasound guidance (Sonosite EDGE, Sonosite Inc., Bothell, WA, USA) with a linear 6–13 MHz transducer, and 15 ml of the drug on each side was given from the medial end of internal oblique abdominis muscle to outward direction (Fig. 1B).

The patients were awakened from anesthesia after TAP block and transferred to PACU. They had their first interview about postoperative wound pain (at rest and during movement) using a 0 to10 point numerical rating scale (NRS) immediately after admission to PACU. After the first interview, fentanyl infusion by intravenous patient-controlled analgesia (iv-PCA) was initiated. An electrical device (CADD Legacy Solis, Smith Medical ASD, Minneapolis, MN, USA) was used for fentanyl infusion with the primary setting of no background infusion, bolus of 20 µg, lockout time of 10 minutes and maximum dose of 100 µg per hour. Other supplemental analgesics could be used after the first interview. Typically, 1000 mg of intravenous acetaminophen was used as the first choice, 50 mg of intravenous flurbiprofen axetil as second. For patients who complained severe pain at the time of awakening from anesthesia, the first interview could be performed before transferred to PACU in order to start fentanyl iv-PCA infusion as soon as possible after we obtained NRS score.

### Data collection

The primary outcome was NRS scores at rest and during movement when the patient was transferred to PACU. NRS data were collected at PACU admission, PACU discharge (about 1 hour after extubation), several hours after the end of surgery at hospital ward (shown as postoperative day (POD) 0 hospital ward) and next day (POD1). All NRS data were collected by anesthesiologists who were not aware of group assignment. Blood pressure and heart rate in PACU were recorded at the time of NRS data collection by on-site anesthesiologists. Patients with unavailable NRS data at PACU admission were excluded from the study. The secondary outcome was postoperative iv-PCA fentanyl dose for the first 24 hours from iv-PCA devices. Use of additional analgesics and complaint of PONV in the first postoperative 24 hours were also obtained. Patient characteristics and the position of anesthesiologists in charge of each patient were also recorded.

### Calculation of the number of required patients

Power analysis was carried out before initiation of this study with the assumption of a normal distribution. From retrospective data of RALP performed only by general anesthesia prior to this study, mean and standard deviation values of NRS at rest when the patients were admitted PACU were 3.7 and 3.3, respectively. Since fentanyl dose limit during surgery (3 µg/kg) was set, we hypothesized that the mean value of NRS at rest in the Control group would be 5 and that there would be 35% decrease in NRS by TAP block. Standard deviation, alpha-error and statistical power were estimated to be 3, 0.05 and 0.8, respectively, 96 patients (48 patients in each group) were required to have significant difference in the conditions shown above. Added 2 patients in each group as margin, we recruited 100 patients (50 patients in each group) for data analysis. A computer-randomized group assignment was made for 100 patients before initiation of this study. Since several patients would be excluded during data collection for various reasons (protocol violation, failed primary outcome, etc.), the number of excluded patients was counted when data collection of the first 60 patients was completed. Then the first even number of excluded patients divided by 0.6 was added without opening group assignment. The additional patients were assigned to either one of two groups in the same manner as that for the first 100 patients.

### Statistical analysis

Statistical analysis was performed using JMP version 12.2 (SAS Institute Inc., Cary NC, USA). According to the results of normal distribution analysis, blood pressure and heart rate were shown as mean ± standard deviations, and others were shown as median (interquartile range). Student’s or Welch’s t-test (parametric data) or Wilcoxon’s test (nonparametric data) was used for statistical analysis. Position of anesthesiologists in charge of patients, the number of patients who had additional analgesics and complained PONV were analyzed using the chi-square test. In all settings, P < 0.05 considered to be a significant difference.

## Results

**Figure 2:**
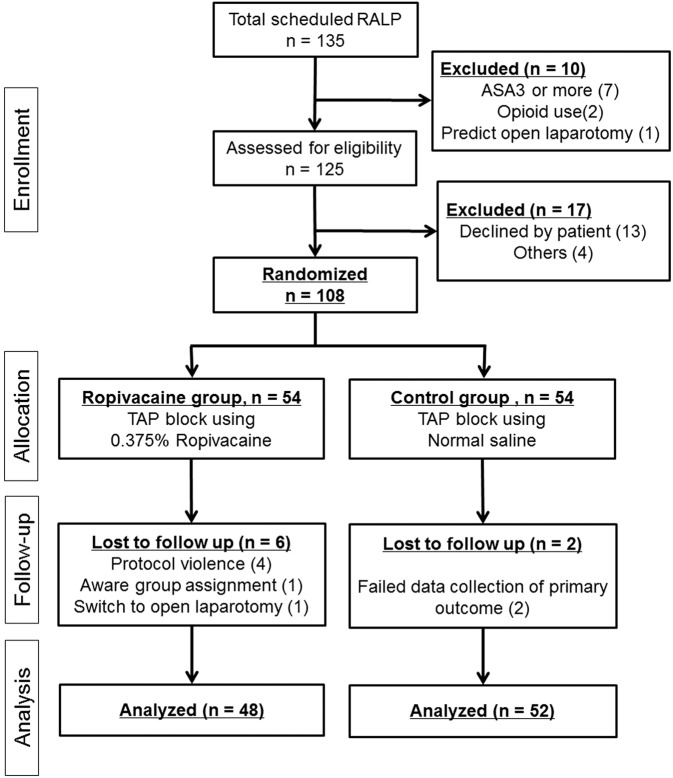
CONSORT flow diagram. RALP, robot-assisted laparoscopic prostatectomy; ASA, American Society of Anesthesiologists; TAP, transversus abdominis plane.

This study was conducted from March 2017 to March 2018. A total of 135 patients were scheduled for RALP, and 125 patients were eligible for this study. The study flow is shown in Fig. 2. Four patients were excluded during study procedures when the data collection of 60 patients had been completed (2 lacking the primary outcome, 1 accidentally aware group assignment and 1 protocol violation). Therefore, eight patients (first even number above 4 divided by 0.6) were added (4 patients assigned to each group). As a result, 108 patients were enrolled in this study. Four additional patients were excluded after this addition (3 protocol violation, 1 switch to laparotomy), data from 100 patients were advanced for assessment. Since group assignment was open after final data acquisition, 48 cases were assigned to Ropivacaine group and 52 cases were assigned to Control group.

There was no significant difference between the two groups in patient characteristics (Table 1). A total of 34 anesthesiologists (5 faculties, 12 fellows and 17 residents) were involved and more than half of TAP blocks were performed by residents in anesthesia. We did not detect any significant differences in the position of anesthesiologists in charge and all perioperative variables (Table 1).

**Table 1:** Patient characteristics, perioperative variables.

n	Ropivacaine group 48	Control group 52	P value
Age (yrs)	68 [64–72]	70 [66–73]	0.16
Height (cm)	167 [163–171]	166 [163–171]	0.65
Weight (kg)	65 [61–72]	67 [62–74]	0.27
Body mass index (kg/m^2^)	23.9 [21.8–25.3]	24.4 [22.7–26.3]	0.24
**Position of in-charge anesthesiologist**			
Resident/Staff/Faculty	30/11/7	28/13/11	0.62
Operation time (min)	200 [146–231]	197 [166–228]	0.85
Anesthesia time (min)	269 [214–308]	267 [245–310]	0.70
Intraoperative fentanyl dose (µg)	200 [176–200]	200 [179–200]	0.92
(divided body weight (µg/kg))	2.84 [2.58–3.12]	2.80 [2.51–3.07]	0.37
Intraoperative remifentanil dose (µg)	2535 [1951–3630]	2675 [2214–3632]	0.84
Duration from wound infiltration to end of anesthesia (min)	45 [39–50]	49 [40–54]	0.10
Duration from remifentanil terminate to end of anesthesia (min)	35 [30–40]	37 [29–44]	0.13
Duration from TAP block to NRS interview at PACU admission (min)	27 [24–31]	29 [26–32]	0.14
**Duration from end of anesthesia to NRS interview (min)**			
PACU admission	8 [6–10]	8 [6–11]	0.78
PACU discharge	53 [44–66]	51 [44–59]	0.39
POD0 hospital ward	198 [140–244]	189 [150–232]	0.62
**Systolic blood pressure (mmHg)**			
PACU admission	152 ± 24	152 ± 21	0.95
PACU discharge	144 ± 20	140 ± 20	0.31
**Heart rate (bpm)**			
PACU admission	83 ± 13	80 ± 13	0.38
PACU discharge	77 ± 13	74 ± 11	0.15

NRS, Numerical rating scale; PACU, Post-anesthesia care unit; POD, Postoperative day.

**Figure 3:**
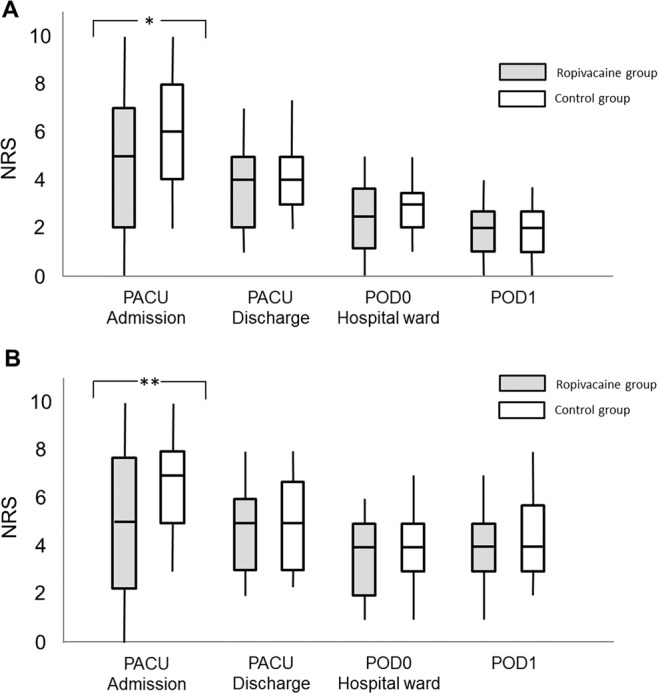
(**A**) NRS at rest. (**B**) NRS during movement. Rectangles show 25 to 75 percentile, and vertical lines show 10 to 90 percentile. Shaded and blank rectangles indicates Ropivacaine group and Control group, respectively. NRS, numerical rating scale; PACU, post-anesthesia care unit; POD, postoperative day. *Means P < 0.05, **means P < 0.01.

NRS at rest is shown in Fig. 3A. Median values of NRS in Ropivacaine group and Control group when patients were transferred to PACU were 5 [2–7] and 6 [4–8], respectively, and the difference was significant (P = 0.03). This significant difference disappeared at the time of PACU discharge (median values in Ropivacaine group and Control group: 4 [2–5] and 4 [3–5], respectively (P = 0.72)). Median values of NRS at POD0 hospital ward and POD1 were 3 or less in both groups (P values: 0.64 and 0.78, respectively).

NRS during movement is shown in Fig. 3B. Median values of NRS at the time of PACU admission in Ropivacaine group and Control group were 5 [2–8] and 7 [5–8] with significant difference (P < 0.01). This significant difference disappeared at the time of PACU discharge (median values being 5 in both groups (P = 0.60)). Median values of NRS during movement in the two groups were 4 at POD0 hospital ward and POD1 (P values: 0.41 and 0.85, respectively).

Total iv-PCA fentanyl doses for 1 hour, 4 hours, 8 hours and 24 hours after the end of anesthesia are shown in Table 2. Although the 25–75 percentiles were lower in Ropivacaine group, we did not detect significant differences between the two groups in any of the time settings. The number of patients who had additional analgesics in PACU tended to be smaller in Ropivacaine group (15 patients, 31% of Ropivacaine group patients) than in Control group (23 patients, 44% of Control group patients) without significant difference (P = 0.18). We could not detect any differences between the two groups in frequency of use of supplemental analgesics for the first 24 postoperative hours (Table 2). We did not detect any significant differences between the two groups in PONV (Table 2). No patient with any adverse effect of TAP block such as bleeding, paresthesia and symptom of local anesthetics systemic toxicity was recorded. Although two patients (one patient in each group) prolonged hospital stay caused by anastomotic leakage which was not related to TAP block performance, other 98 patients kept inpatient perioperative pathway (removal of urine catheter and duration of hospital stay).

**Table 2:** Postoperative fentanyl and supplemental analgesics, postoperative nausea and vomiting.

n	Ropivacaine group 48	Control group 52	*P* value
iv-PCA fentanyl dose			
Duration from end of anesthesia			
~1 h	40 [20–60]	40 [25–80]	0.09
~4 h	80 [20–120]	100 [60–135]	0.12
~8 h	100 [40–160]	120 [60–175]	0.24
~24 h	210 [120–360]	200 [120–370]	
Frequency of supplemental analgesics			
~24 h total	1 [1,2]	1 [0–3]	0.81
(after discharging PACU)	1 [0–2]	1 [0–2]	0.48
Number of Patients with PONV in the first postoperative 24 hours	15/48 (31.3%)	15/52 (28.9%)	0.79
Use antiemetics	10/48 (20.8%)	12/52 (23.1%)	0.79

iv-PCA, intravenous patient-controlled analgesia; PACU, Post-anesthesia care unit;

PONV, Postoperative nausea and vomiting.

## Discussion

The major finding of our study is that a subcostal TAP block after the RALP procedure reduced the 11-point NRS at rest and during movement when the patients were admitted to PACU.

Recent randomized controlled studies (2011 ~ current) on the analgesic effect of single-shot TAP block after laparoscopic lower abdominal surgery are shown (Table 3)^7–10,12–19^. Although a meta-analysis including these investigations suggested that TAP block reduces perioperative opioid consumption^20^, NRS was decreased in only 4 of those 12 studies. We speculated that apparent analgesic effect of TAP block might have affected by methodological factors such as block approach, block timing, and use of opioids or supplemental analgesics during surgery. Therefore, we gave the greatest consideration to maximize analgesic effect of TAP block when we designed our protocol.

**Table 3:** Recent double-blind trials on analgesic effect of TAP block for lower abdominal laparoscopic surgery (later than 2011).

	Deolivera (2011)^9^	Kane (2012)^12^	Walter (2013)^13^	Calle (2014)^14^	Keller (2014)^7^	Smith (2015)^15^
**Surgery**	**LAH**	**LAH**	**LAC**	**LAH**	**LAC**	**LAC**
Group (Intervention/Control)	0.25 R, 0.5 R/NS	0.5%R/No sham	0.25LB /No sham	1.5 mg/kg B/NS	0.5B/NS	3 mg/kg R /no sham
Sample size (Intervention/Control)	21, 22/23	28/29	33/35	100/97	41/38	68/74
Block timing	preoperative	postoperative	preoperative	before closure	before closure	preoperative
Anesthesiologist	***	Pain management team	>20 TAP experienced	block by surgeon	block by surgeon	***
Surgeon	***	***	***	sole surgeon	4 experienced	3 operator
Intraoperative opioids	hydromorphine	***	morphine	***	***	***
Intraoperative supplemental analgesics	30 mg Ketorolac	30 mg Ketorolac	***	***	***	***
Assessment (NRS)	up to 24 h	2 and 24 h	2 to 24 h	Discharge,24,48,72 h	PACU, Discharge	24 to 72 h
Assessment (Opioids)	morphine	morphine	morphine	No opioids	morphine	morphine
Result (NRS)	**Positive at PACU**	Negative	Negative	**Positive at discharge**	**Positive**	Negative
Result (Opioids)	**Positive during PACU**	Negative	**Positive for first 24 h**		**Positive at PACU**	Negative
	Torup (2015)^10^	Ghisi (2016)^16^	Rashiod (2016)^17^	Tikuisis (2016)^18^	Torup (2016)^19^	Oh (2017)^8^
**Surgery**	**LAH**	**LAH**	**LAC**	**LAC**	**LAC**	**LAC**
Group (Intervention/Control)	0.5 R/NS	0.375 R /no sham	0.25B /Wound infiltration	0.375 R/NS	0.5 R/NS	0.25B/NS
Sample size (Intervention/Control)	34/31	22/22	28/28	32/32	40/40	28/27
Block timing	preoperative	preoperative	preoperative	preoperative	preoperative	preoperative
Anesthesiologist	4 experienced	***	3 experienced	sole anesthesiologist	4 experienced	>5 yr experience
Surgeon	***	sole surgeon	3 operator	***	***	***
Intraoperative opioids	0.2 mg/kg morphine 45 min before end	remifentanil	***	***	1.5 µg/kg fentanyl 45 min before end	***
Intraoperative supplemental analgesics	paracetamol	***	***	***	paracetamol	***
Assessment (NRS)	up to 24 h	PACU, POD0, 1	6,12,24,48 h	2,4,8,12,24 h	up to 24 h	1 h, POD1,2,3
Assessment (Opioids)	morphine	morphine	morphine	***	morphine	Morphine, Fentanyl
Result (NRS)	Negative	Negative	Negative	**Positive up to 4** **h (rest on 12 h)**	Negative	Negative
Result (Opioids)	Negative	Negative	Negative		**Positive at PACU, 24 h**	Negative

LAH, Laparoscope assisted hysterectomy; LAC, Laparoscope assisted colectomy; R, Ropivacaine; LB, Levobupivacaine;

B, Bupivacaine; NS, 0.9% saline; PACU, Post-anesthesia care unit; POD, Postoperative days; ***no statement about the column. ‘Positive’ indicates that intervention groups had significant analgesic effect than that in control groups.

In RALP, analgesia of camera port scar (Fig. 1A) requires the Th9 dermatome area. Local anesthetics in the original TAP block may reach the Th7 dermatome area^21^ but needs a half lateral position. In a mid-axillary approach, which is the most popular, local anesthetics would not reach the Th9 dermatome area^22^. Since half of the Th9 nerves were shown to be involved by single-shot dye injection in a cadaveric study^23^ and we could perform without removing surgical drapes, a subcostal approach was selected in our study. Our approach is almost the same as reported by Carney *et al*.^24^ to minimize the variation in injection sites among anesthesiologists. Further, we did not put restrictions on practitioners performing TAP blocks. In many studies in which the effect of intervention was evaluated, the number of practitioners was restricted to minimize the skill-based bias between practitioners. This restriction may maximize the effects of intervention, but reproducibility is unclear. In fact, specific skilled surgeons or anesthesiologists performed TAP blocks in 3 of 4 recent studies in which TAP block had beneficial effects on postoperative NRS^7,14,18^. In our study, 34 anesthesiologists including residents performed TAP blocks, so our positive analgesic effect by TAP blocks should be universal regardless of the experience of the practitioner.

TAP blocks were performed postoperatively to equalize the duration between TAP blocks to the first interview (25~30 minutes, Table 1) and to maximize plasma concentration of ropivacaine at the first interview^25,26^. Also, we did not allow any supplemental analgesics other than remifentanil and a limited dose of fentanyl during surgery in order to eliminate analgesic effects other than TAP block. As shown in method section, we asked surgeons to give 10 ml of 0.2% ropivacaine to camera port and did not allow intravenous fentanyl after this wound infiltration. In our study, 74 patients (36 (75%) in Ropivacaine group and 38 (73%) in Control group) received total dose of intravenous fentanyl less than 30 minutes before wound infiltration. So we simulated fentanyl and remifentanil concentrations at the end of anesthesia, assuming 3 µg/kg of fentanyl would be given as wound infiltration and that continuous remifentanil would be infused from the induction of anesthesia to the end of surgery (approximately 0.17 µg/kg/min, median cumulative dose at a flat rate), for patients with median characteristics using anesthesia record software (Prescient® OR, Fujifilm Corporation, Tokyo, Japan)^27,28^. The simulated plasma remifentanil concentrations were below minimum effective concentration. The simulated plasma fentanyl concentrations in the Ropivacaine and Control groups were 0.70 ng/ml and 0.63 ng/ml, respectively, suggested that minimal analgesia should be provided by fentanyl^29^. Due to our special considerations including setting injection site, performing TAP blocks postoperatively and limiting supplemental analgesia during surgery, an inherent analgesic effect of TAP block was revealed. Significant analgesic effect of TAP block at the time of PACU admission was enlarged in patients aged 65 years and elder (Median values of NRS in Ropivacaine group (34 patients) vs. Control group (41 patients): 4 [1–5] vs. 5 [4–8] (P < 0.01),), and was missing in the patients less than 65 years old (25 patients) (P = 0.76).

Our results also showed the fragility of analgesic effect of TAP block. Only two bolus requests for iv-PCA fentanyl (40 µg) in PACU erased the significant analgesic benefit of TAP block. Furthermore, median NRS at rest in the Ropivacaine group at PACU admission was 5 (estimated value in the Control group). There are some possible factors affecting these insufficiencies. First, we had to restrict total ropivacaine dose to 132.5 mg (112.5 mg for TAP block and 20 mg for wound infiltration), which was about 3 mg/kg for patients weighing 45 kg, because of the ethical reason to keep blood ropivacaine concentration in therapeutic range^26^. This restricted dose would meet minimum requirement^30^ but was relatively small for the patient with median characteristics (approximately 2 mg/kg for patients weighing 66 kg). Analgesic effect of TAP blocks may be clear when we set 3 mg/kg of ropivacaine for each patient. Second, we asked practitioners to perform ‘single injection‘ subcostal TAP blocks which would cover half of the Th9 dermatome area^23^. Our priority was to avoid restriction on practitioners performing TAP blocks, but this priority let us make another restriction about needle injection site for minimizing variation between practitioners^24^. If we allow practitioners to perform ‘multi-injection‘ subcostal TAP blocks, spread of local anesthetics behind rectus sheath (cover 86% of the Th9 dermatome area)^23^ may result better pain scores. Third, overall NRS scores would be elevated because of discomfort for bladder-urinary tract anastomosis or urine catheter by limiting intravenous fentanyl during surgery. However, all patients received same anesthetic protocol except for TAP block drugs, these discomforts should not affect analgesic effect of TAP blocks.

In summary, ultrasound-guided, single-shot TAP block by a subcostal approach reduced early postoperative pain after robot-assisted laparoscopic prostatectomy (RALP) by anesthesiologists with various levels of training. However, analgesic effect of TAP block was small and disappeared by supplemental patient-controlled intravenous fentanyl in PACU and did not affect the first postoperative 24-hour fentanyl consumption. Our results indicated that TAP block has fundamental analgesic effect but may not able to provide satisfiable analgesia solely. We should consider TAP blocks as one of various options in perioperative multimodal analgesia after RALP.

### Presentation

Data for this study was presented at American Society of Regional Anesthesia annual meeting (April 2019), Las Vegas, United States.
